# A Network Pharmacology and Molecular-Docking-Based Approach to Identify the Probable Targets of Short-Chain Fatty-Acid-Producing Microbial Metabolites against Kidney Cancer and Inflammation

**DOI:** 10.3390/biom13111678

**Published:** 2023-11-20

**Authors:** Md. Rezaul Karim, Md. Niaj Morshed, Safia Iqbal, Shahnawaz Mohammad, Ramya Mathiyalagan, Deok Chun Yang, Yeon Ju Kim, Joon Hyun Song, Dong Uk Yang

**Affiliations:** 1 Department of Biopharmaceutical Biotechnology, College of Life Science, Kyung Hee University, Yongin-si 17104, Republic of Korea; rezaulshimul@khu.ac.kr (M.R.K.); niajmorshed96@khu.ac.kr (M.N.M.); safiadorin@khu.ac.kr (S.I.); dcyang@khu.ac.kr (D.C.Y.); 2 Department of Biotechnology and Genetic Engineering, Faculty of Biological Sciences, Islamic University, Kushtia 7003, Bangladesh; 3 Department of Microbiology, Varendra Institute of Biosciences, Affiliated University of Rajshahi, Natore, Rajshahi 6400, Bangladesh; 4 Graduate School of Biotechnology, College of Life Science, Kyung Hee University, Yongin-si 17104, Republic of Korea; shnwzmohd@yahoo.com (S.M.); ramyabinfo@gmail.com (R.M.); yeonjukim@khu.ac.kr (Y.J.K.); 5 Hanbangbio Inc., Yongin-si 17104, Republic of Korea; 6 Department of Veterinary International Medicine, College of Veterinary Medicine, Chungnam National University, Daejeon 34134, Republic of Korea; 7 AIBIOME, 6, Jeonmin-ro 30beon-gil, Yuseong-gu 34052, Republic of Korea

**Keywords:** network pharmacology, gut microbial metabolites, SCFA-producing microbes, kidney cancer, kidney inflammation, molecular docking

## Abstract

(1) Background: A large and diverse microbial population exists in the human intestinal tract, which supports gut homeostasis and the health of the host. Short-chain fatty acid (SCFA)-secreting microbes also generate several metabolites with favorable regulatory effects on various malignancies and immunological inflammations. The involvement of intestinal SCFAs in kidney diseases, such as various kidney malignancies and inflammations, has emerged as a fascinating area of study in recent years. However, the mechanisms of SCFAs and other metabolites produced by SCFA-producing bacteria against kidney cancer and inflammation have not yet been investigated. (2) Methods: We considered 177 different SCFA-producing microbial species and 114 metabolites from the gutMgene database. Further, we used different online-based database platforms to predict 1890 gene targets associated with metabolites. Moreover, DisGeNET, OMIM, and Genecard databases were used to consider 13,104 disease-related gene targets. We used a Venn diagram and various protein−protein interactions (PPIs), KEGG pathways, and GO analyses for the functional analysis of gene targets. Moreover, the subnetwork of protein−protein interactions (through string and cytoscape platforms) was used to select the top 20% of gene targets through degree centrality, betweenness centrality, and closeness centrality. To screen the possible candidate compounds, we performed an analysis of the ADMET (absorption, distribution, metabolism, excretion, and toxicity) properties of metabolites and then found the best binding affinity using molecular docking simulation. (3) Results: Finally, we found the key gene targets that interact with suitable compounds and function against kidney cancer and inflammation, such as MTOR (with glycocholic acid), PIK3CA (with 11-methoxycurvularin, glycocholic acid, and isoquercitrin), IL6 (with isoquercitrin), PTGS2 (with isoquercitrin), and IGF1R (with 2-amino-1-methyl-6-phenylimidazo[4,5-b] pyridine, isoquercitrin), showed a lower binding affinity. (4) Conclusions: This study provides evidence to support the positive effects of SCFA-producing microbial metabolites that function against kidney cancer and inflammation and makes integrative research proposals that may be used to guide future studies.

## 1. Introduction

As kidney cancer causes more than 131,000 fatalities and 342,000 incident cases worldwide each year, it is one of the most serious malignancies [1]. According to 2020 GLOBOCAN data, globally, 2.2% of total incidences and 1.8% of total cancer deaths occur in kidney cancer annually [2,3]. Cancer research has recently shifted its attention to the link between cancer and inflammation [4]. Numerous studies have demonstrated that the development of cancer, including breast, pancreatic, colorectal, colon, rectal, prostate, bladder, lung, and ovarian cancers, is strongly influenced by inflammatory chemicals and pathways [5,6]. Renal cell carcinoma (RCC) and inflammation are both closely related, and both contribute to the growth of RCC tumors, which are thought to be immunogenic [7,8]. Surgery is still the most effective treatment for both localized and locally progressed RCC, because 25–30% of affected patients have metastatic disease and, therefore, a poor prognosis [9]. Therefore, the current study focuses on inflammation and kidney cancer. We focused on network pharmacology analysis to find the safer and more effective therapeutic gene targets needed to treat inflammation and kidney cancer.

In recent years, there has been growing interest in the gut−kidney interaction as it relates to chronic kidney disease (CKD), including kidney cancer and inflammation [10]. Microbial metabolites can function as signaling substances when circulated throughout the body [11]. Currently, short-chain fatty acids (SCFAs) and their receptors, as well as changes to the gut microbiome, are some of the suggested mechanisms linking dysbiotic gut microbiota to CKD and its consequences [11,12,13]. As a class of metabolites, SCFAs exert advantageous regulatory effects on blood pressure, immunological inflammation, hormone production, and cancer [14]. A lack of gut-microbiota-produced SCFAs has also been linked to disorders such as inflammatory bowel disease, obesity, type 1 and type 2 diabetes, autism, major depression, colon cancer, and renal diseases, which are the topic of this discussion [15,16,17,18]. However, the relationship between bacteria that produce SCFAs and the various metabolites they secrete, including SCFAs, which help treat kidney inflammation and kidney cancer, is still not entirely understood. Therefore, the current study focuses on microbes that produce SCFAs and their metabolites to find potential targets for the treatment of inflammation and kidney cancer using network pharmacology.

To combat the co-morbidity of these diseases, the goal of this research was to discover the most significant gut SCFAs-producing microbial compounds that can be used to control the expression of the hierarchical targets for treating kidney cancer and inflammation. We also discussed the important SCFAs-producing probiotics that the molecular docking test (MDT) determined to be the most stably bound metabolites on a significant target. As a result, our research may identify approaches to reduce kidney cancer and inflammation by using the effect of complex microbiome networks.

## 2. Materials and Methods

### 2.1. Target Gene Prediction of SCFAs Microbial Metabolites and Diseases (Kidney Cancer and Inflammation)

The targets and metabolites of the gut SCFA-producing microbiota were obtained using the gutMGene v1.0 database (http://bio-annotation.cn/gutmgene/) (accessed on 10 July 2023) [19]. To retrieve kidney cancer and inflammation-related targets, we used the DisgeNET v7.0(https://www.disgenet.org/) (accessed on 12 July 2023) [20], OMIM (https://www.omim.org/) [21] (accessed on 13 July 2023), and Genecard databases v5.8 (https://www.genecards.org/) (accessed on 14 July 2023) [22] databases. The major targets among the metabolites and chosen disease-related targets were identified using Venny 2.1.0 (https://bioinfogp.cnb.csic.es/tools/venny/ accessed on 15 July 2023), an online mapping tool. We used the overlapped genes for further analysis using GeneMANIA (https://genemania.org/ V3.6.0 version accessed on 16 July 2023) [23] to find the target genes that are co-expressed and that share the same protein and pathway. We then used those genes for further analysis.

### 2.2. Target Gene Location in Chromosomes and Tissues

To evaluate the pathophysiology of some genes and identify the potential therapeutic targets, the chromosomal location of the target genes needs to be determined. As a result, the location of the genes on the chromosomes was determined using the ShinyGO web tool v0.75 (http://bioinformatics.sdstate.edu/go/ accessed on 18 July 2023) [24]. Additionally, the distribution of shared genes varied in other organs. So, using the Pa-GenBase dataset from the Metascape web server v3.5.2023.05.01 (https://metascape.org/gp/index.html#/main/ accessed on 19 July 2023) [25], we were able to determine the distribution of genes that affect different tissues and cell types.

### 2.3. Analysis of Target Gene Pathways Using Gene Ontology (GO) and Kyoto Encyclopedia of Genes and Genomes (KEGG) Databases

GO analysis, which was created to define the activities of the targets, included analyses of cellular components (CC), biological function, and molecular function. The KEGG pathway analysis shed light on the putative signaling pathways linked to the final targets against kidney cancer and renal inflammation. The gene ratio of the differentially expressed genes to the total number of targets in a signaling pathway serves as the foundation for enrichment plots, which are based on a *p*-value and adj. *p*-value [26]. Gene ontology and pathway studies were performed using the Protein Analysis Through Evolutionary Relationships (PANTHER) program v18.0 (http://pantherdb.org/ accessed on 20 July 2023) to determine how these frequently connected genes collectively influence the signaling pathways [27].

### 2.4. Protein−Protein Interaction (PPI) Network Analysis of Targeted Gene

Protein interactions are examined throughout the early stages of drug development because they provide an immense amount of information about the functions of proteins [28]. The total number of intricate biological processes is estimated through a thorough PPI network investigation [29]. By using the STRING dataset with NetworkAnalyst v3.0 (https://www.networkanalyst.ca/ accessed on 22 July 2023), the PPI of common genes has been determined to examine the molecular mechanisms linked to major signaling pathways and cellular activities [30]. The PPI network was created using the fundamental PPI configuration, which used H. sapiens as the organism, STRING as the database, and a confidence score cutoff of 900. After assessing the accuracy, we concluded that the common nodes were the most likely hubs. The creation of sub-PPI networks using CytoHubba v0.1 in Cytoscape version 3.10.1 to select the suitable gene targets using the highest degree centrality (DC) values, highest betweenness centrality (BC) values, and highest closeness centrality (CC) values in the top 20% of the PPI networks [31].

### 2.5. Analysis of the Physiochemical and ADMET Characteristics of Microbial Compounds

The hit compounds were subjected to in silico physiochemical analysis. The SwissADME (http://www.swissadme.ch/index.php accessed on 23–28 July 2023) [32], ADMETlab2.0 (https://admetmesh.scbdd.com/ accessed on 23–28 July 2023) [33], and pkCSM (https://biosig.lab.uq.edu.au/pkcsm/ accessed on 23–28 July 2023) web servers were used for the drug-likeness analysis [34], and the Protox-II web server (https://tox-new.charite.de/protox_II/ accessed on 23–28 July 2023) was used for the analysis of the predicted ADMET properties [35]. To predict ADMET properties (absorption, distribution, metabolism, excretion, and toxicity), the PubChem Database’s canonical SMILES of the metabolites were consulted. Finally, we rejected the protein targets connected to Lipinski’s rule of five (LO5) metabolites that broke more than two of its rules [36]. Investigations were conducted using the remaining targets and metabolites.

### 2.6. Validation of the Expression of the Hub Targets

Information on the expression and distribution of various human proteins in diverse tissues is made available via the Human Protein Atlas database (HPA) v23.0 (https://www.proteinatlas.org/ accessed on 2 August 2023) [37]. Using data from the Human Protein Atlas, we investigated the expression levels of hub targets in the kidneys and urinary bladder.

### 2.7. Protein and Ligand Preparation

From the RCSB protein data bank (www.rcsb.org, accessed on 1 August 2023), the crystal structures of MTOR (PDB:2FAP), PIK3CA (PDB:5DXT), IL6 (PDB:4ZST), PTGS2 (PDB:5IKQ), and IGF1R (PDB:5FXQ) were retrieved [38]. All of the retrieved protein structures belonged to the human database. For the selection of protein structures, we considered mainly the X-ray diffraction experimental method, and also the refinement resolution range between approximately 1.5 and 2.5. The proteins were prepared by removing the cofactors, water molecules, and metal ions from the complex structure. After the non-polar hydrogen atoms were combined and polar hydrogen atoms were added, Gasteiger charges for the protein were computed [39]. The aromatic carbons were located, the non-polar hydrogens were combined, and the molecule ‘torsion tree’ was set up using AutoDock v4 Tools. For additional screening, the PDBQT file format of these results was used. Additionally, the PubChem Database (https://pubchem.ncbi.nlm.nih.gov/ accessed on 14–20 August 2023) was utilized to acquire the 3D structures of the active components [40]. Finally, SDF files were used as the download format.

### 2.8. Binding Site Identification and Grid Box Generation 

Binding sites were found by comparing pockets from known protein−ligand interactions. PDB and CASTp (http://sts.bioe.uic.edu/castp/ accessed on 14–20 August 2023) [41] were used to extract the known and unknown active sites of the protein structures, respectively, and BIOVIA Discovery Studio Visualizer v19.1 (BIOVIA) was used to examine the binding site of the proteins [42]. The receptor grid was built using molecular docking and the binding sites were obtained from the complex structure using the PyRx—Python Prescription 0.8 virtual screening tool [43].

### 2.9. Molecular Docking Simulation

A molecular docking simulation was performed using the PyRx v0.8 tool to identify the candidates that were most compatible with the target proteins [30]. AutoDock Vina and AutoDock v4 are included in PyRx, a free computational screening application that can assess a big dataset against a specific biologically targeted macromolecule. The default setting in PyRx v0.8 is the AutoDock Vina Wizard v4 [44], which simulates molecular docking. In comparison with other compounds, the top compounds had the highest binding affinity (kcal/mol) to the target protein. Finally, using the default arrangement, receptor grids were created.

## 3. Results

### 3.1. Retrieve Metabolites and Potential Target Proteins Linked to Kidney Cancer and Kidney Inflammation

**Figure 1:**
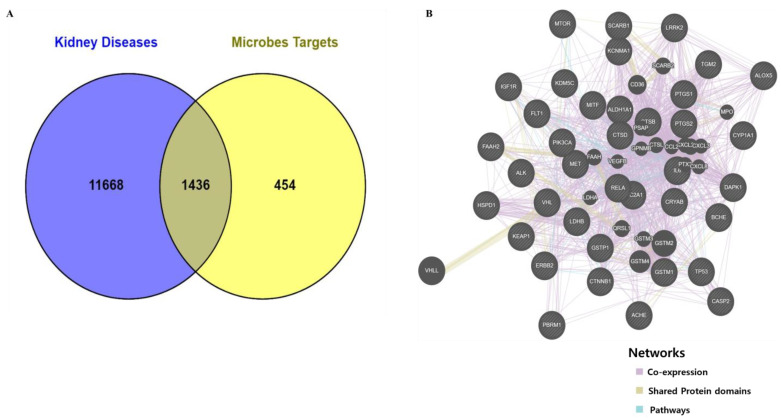
(**A**) Venn diagram analysis between metabolites and disease-related targets. (**B**) GeneMANIA analysis to predict co-expressed, same protein domain, and pathway targets.

We obtained 177 SCFA-producing microbes and 114 metabolites from the gutMgene microbiome database. A total of 1890 metabolite-related gene targets were predicted from the gutMgene, PubChem, and Human Metabolome (HMDB) databases, using the similarity ensemble approach (SEA), Swiss target prediction (STP), and Chemical Entities of Biological Interest (ChEBI) (Supplementary S1). A total of 13,104 target genes for kidney cancer and kidney inflammation-related diseases were retrieved from DisgeNET, genecard, and OMIM (Supplementary S2). The revealed targets and compounds were regarded as important factors for analyzing the treatment outcomes of the gut microbiota. Subsequently, 1436 overlapping targets related to kidney cancer and inflammation were identified through a Venn diagram (Figure 1A). Therefore, the 1436 selected targets were used for further analysis in GeneMANIA to find co-expressed genes that shared the same protein domain and related gene regulation pathways. Finally, we found 38 gene targets that were used for further analysis (Figure 1B, Supplementary S3).

### 3.2. Distribution and Location of Genes

**Figure 2:**
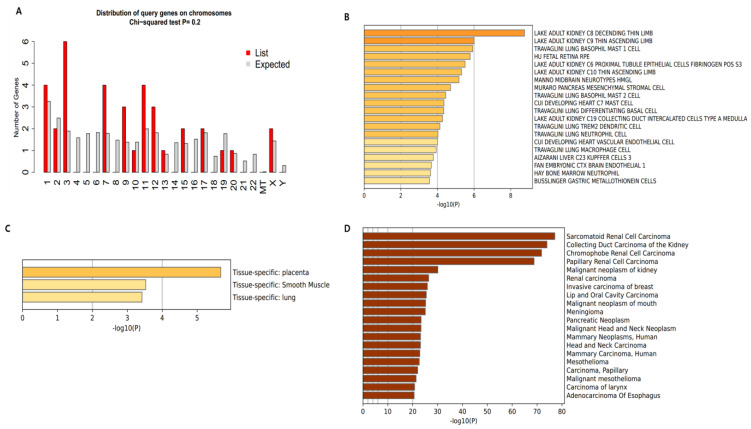
Distribution of targeted genea. (**A**) Chromosomal distribution. (**B**) Cellular distribution. (**C**) Tissue-specific distribution. (**D**) Distribution in different cancer types.

Identifying the exact cellular and molecular locations of the genes is required to identify a protein at the transcription level. We used 38 final targets in the Metascape online server for this analysis. Most of the target genes of kidney cancer and kidney inflammation were expressed in the placenta (expressed value around 6), while others were also expressed in smooth muscle (above 3.5) and in the lungs (above 3) (Figure 2C). Additionally, in the analysis of kidney cancer and inflammation genes, the majority of the common target genes (six) were present in chromosome 3, and four genes were present in chromosomes 1, 7, and 11. Except for the 4, 5, 6, 8, 14, 16, 18, 21, 22, X, and Y chromosomes, the rest were evenly distributed throughout the genome (Figure 1A). As a cellular level, most of the genes were expressed (above 8) in lake adult kidney C8 descending thin limb, and were also highly expressed (around six) in the lake adult kidney C9 thin ascending limb, as well as the travaglini lung basophil mast 1 cell (near to six). In the other kidney and lung cells, the genes were thoroughly distributed (Figure 2B). Finally, we also analyzed and observed that most of the genes were expressed in different renal cell carcinomas and kidney carcinomas (Figure 2D).

### 3.3. Gene Ontology and Pathway Analysis of Gene Targets

**Figure 3:**
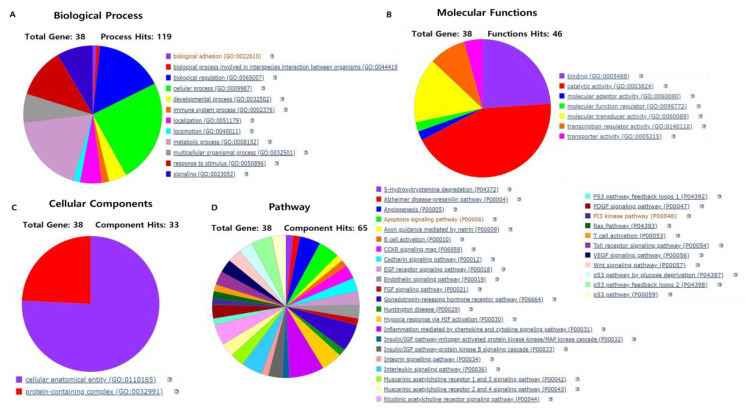
Gene ontology (GO) and KEGG pathway analysis of the target genes. (**A**) Biological process. (**B**) Molecular functions. (**C**) Cellular components. (**D**) KEGG pathway analysis.

To further investigate the potential mechanism of the genes for kidney cancer and inflammation, GO and KEGG enrichment analyses were conducted based on 38 targets. We found these genes enriched in 119 biological process (BP) terms, 46 molecular functions (MF) terms, and 33 cellular component (CC) terms for kidney cancer and inflammation. The top 12 entries from BP, 7 entries from MF, and 2 entries from CC terms are shown in Figure 3 (Supplementary S5). In the case of kidney cancer and inflammation, BP analysis showed that associated targets were primarily centered on biological regulation and cellular and metabolic processes (Figure 3A). According to the MF analysis, potential kidney cancer and inflammation targets were determined mainly based on the binding affinity, catalytic activity, and molecular transducer activity. CC analysis indicated that related targets were primarily mainly centered on the cellular anatomical entity and protein-containing complex (Figure 3C). KEGG pathway enrichment analysis was also performed to investigate the pathways associated with the key targets. The results showed 65 significantly enriched signaling pathways for kidney cancer and inflammation. The top 32 significantly enriched pathways displayed in Figure 3D were closely correlated to kidney cancer and inflammation (Figure 3D). So, the pathway enrichment analysis indicated that the gonadotropin-releasing hormone receptor pathway, inflammation-mediated chemokine and cytokine signaling pathway, angiogenesis and apoptosis signaling pathway, endothelin signaling pathway, interleukin signaling pathway, and nicotinic acetylcholine receptor signaling pathway were most closely related to kidney cancer and inflammation (Figure 3D) (Supplementary S4).

### 3.4. Screening of Hub Targets and PPI Network Construction

**Figure 4:**
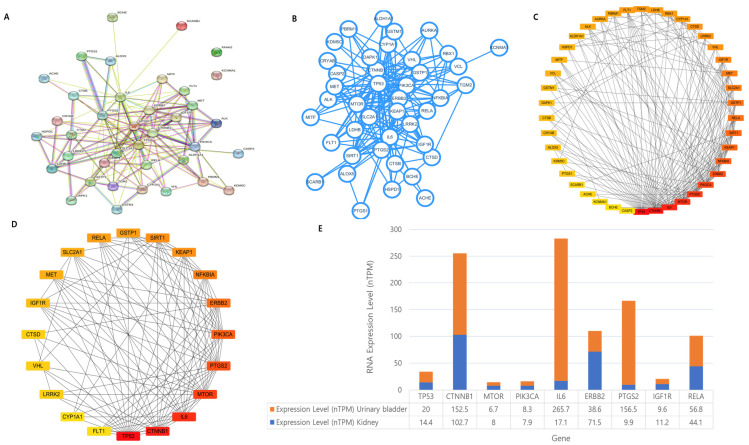
Hub targets and PPI network construction. (**A**) Identification of kidney cancer and inflammation hub proteins. (**B**) PPI network analysis. (**C**,**D**) PPI network of kidney cancer and inflammation-related hub protein, and screening of the top 20 targets based on the degree of centrality (the nodes represent proteins, and the edges represent protein–protein interactions). (**E**) RNA tissue specificity expression profile in the urinary bladder and whole kidneys.

In the PPI network analysis, we used 38 targets. Among these targets, the kidney cancer and inflammation targets FAAH2 and KCNMA1 did not interact with other targets that consisted of 38 nodes and 150 edges (Figure 4A). For further screening, we considered 78 nodes and 640 edges and selected the top 20% of targets based on the degree of centrality (DC). We also checked betweenness centrality (BC) and closeness centrality (CC) (Supplementary S6). Subsequently, we found the top 20 targets that were related to kidney cancer and inflammation (Figure 4B–D) (Table 1). Furthermore, we selected the common targets among the 38 common targets of SCFA-producing microbes and kidney cancer and inflammation, and the top 20 PPI hub targets of kidney cancer and inflammation. Finally, we found nine hub targets (TP53, CTNNB1, MTOR, PIK3CA, IL6, ERBB2, PTGS2, IGF1R, and RELA) related to kidney cancer and inflammation. For additional conformation, we checked the RNA expression of the nine final target genes in the organs of the urinary bladder and whole kidneys. In this analysis, we found a positive expression in both the urinary bladder and whole kidneys (Figure 4E). Further analysis proceeded with the nine final targets, which were related to kidney cancer and inflammation.

### 3.5. Physiochemical and ADMET Property Analysis of Lead Compounds That Control the Hub Targets Expression

After the PPI network analysis, we recovered nine hub targets for kidney disease and inflammation that could be regulated by gut SCFA-producing microbial metabolites (Table 2) (Supplementary S7). The physiochemical and ADMET (absorption, distribution, metabolism, excretion, and toxicity) features of these target-regulated metabolites (75 metabolites) were examined. After analyzing the ADME properties, we screened and rejected some metabolites that did not meet the drug properties (Supplementary S7). Finally, we found metabolites that regulated the nine hub targets (Table 2), that mainly determined by its pharmacodynamics (PD) and pharmacokinetics (PK) properties.

As per Lipinski’s rule of five, an orally administrated drug must have HBA ≤ 10, log P ≤ 5, HBD ≤ 5, and molecular weight (MW) < 500 Daltons [45]. Due to the violation of Lipinski’s rules, we rejected (20S)-protopanaxadiol, aglycone, beta-D-Gal-(1->4)-beta-D-GlcNAc-(1->3)-beta-D-Gal-(1->4)-D-Glc, and ginsenoside Rh2, as well as because of the violation of ADME properties, such as the plasma-protein binding property. The level of plasma-protein binding impacts the drug’s effectiveness, clearance, and possible interactions. Only the unbound fraction of the drug is prone to clearance from the liver and is available for binding to the molecular target [46]. As such, we rejected molecular candidates that did not have plasma-protein binding values below <90%. Candidates rejected based on the criterion included 10-keto-12Z-octadecenoic acid, 2,3-bis(3,4-dihydroxybenzyl) butyrolactone, 6,7,4′,rihydroxyisoflavone, 6′-hydroxy-O-desmethylangolensin, 8-prenylnaringenin, apigenin, arctigenin, chrysin, daidzein, deoxycholic acid, dihydrodaidzein, dihydroglycitein, equol, folic acid, genistein, glycitein, hesperetin dihydrochalcone, kaempferol, naringenin, norathyriol, O-desmethylangolensin, palmitic acid, phloretin, protopanaxadiol, quercetin, and secoisolariciresinol. Finally, we select 9 targets with 41 compounds for further analysis (Table 2, Supplementary S7).

### 3.6. Molecular Docking of a Bioactive Compound with Its Target

Molecular docking was used to examine the molecular interactions between the kidney cancer and inflammation-related targets described above, as well as the SCFAs that produce microbial metabolites. As a control, belzutifan (for kidney cancer) [47] and levofloxacin (for kidney inflammation) [48] were used, and the critical active site residues were flexibly maintained. The findings of the interaction were confirmed by the formation of hydrogen bonds and the binding energy to the necessary active residues and ligands. In molecular docking, we addressed the top nine targets for kidney cancer and inflammation. Supplementary S4 relays information on the selected targets with ligands and the binding affinity. Among all of the molecular docking results, the maximum binding energy was observed to be −2.9 kcal/mol for kidney cancer and inflammation, whereas the minimum binding energy was observed to be –9.5 kcal/mol. After analyzing the obtained binding affinities, we obtained five targets (IGF1R, IL6, MTOR, PIK3CA, and PTGS2) for kidney cancer and inflammation (Table 3). After evaluating the docking results, we predict that 2-amino-1-methyl-6-phenylimidazo (4,5-b) pyridine and isoquercitrin with IGF1R target; isoquercitrin with IL6 target; 11-methoxycurvularin and glycocholic acid with MTOR target; 11-methoxycurvularin, glycocholic acid, and isoquercitrin with PIK3CA target; and isoquercitrin with PTGS2 target showed a lower binding affinity and better stability than the control ligands. 

**Figure 5:**
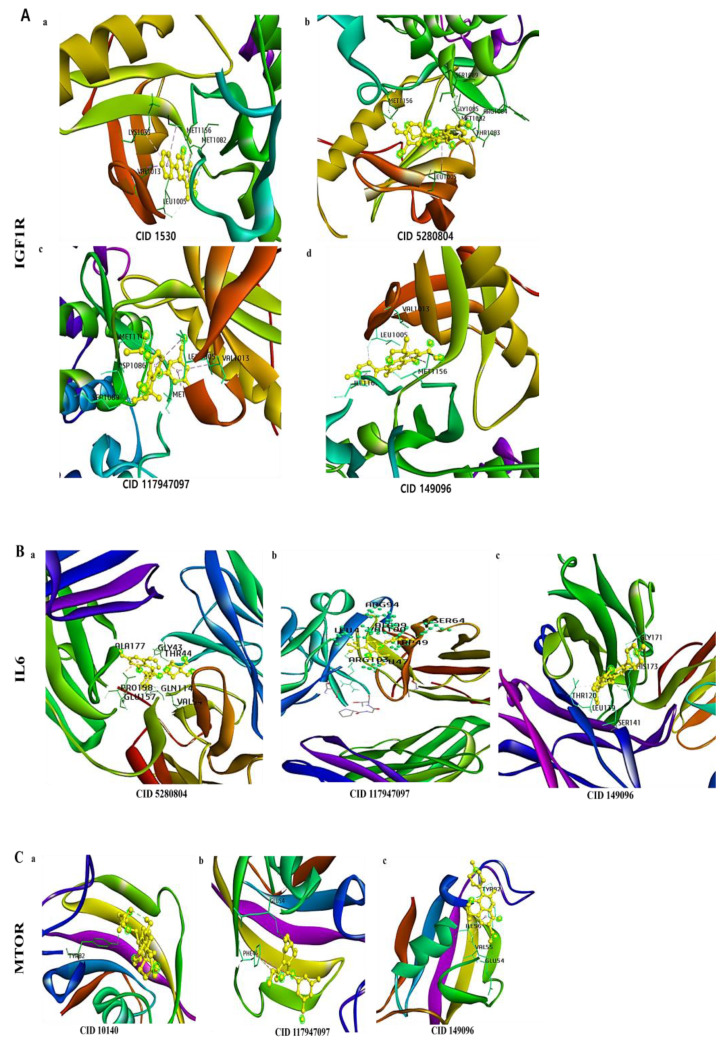

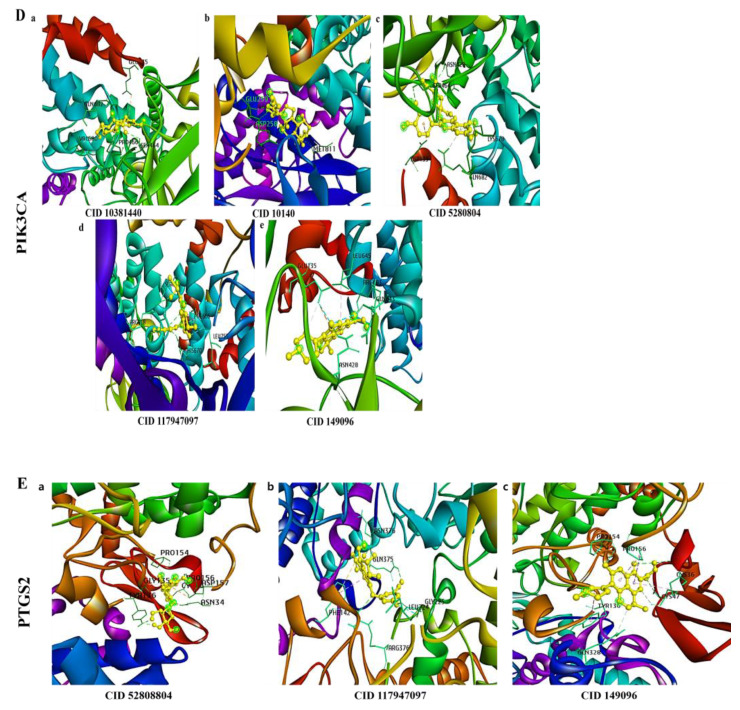
The 3D interactions of kidney cancer and inflammation-related targets with their related SCFA-producing microbial metabolites and control (Belzutifan_ Cancer and Levofloxacin_ inflammation). (**A**) (**a**) IGF1R with 2-amino-1-methyl-6-phenylimidazo(4,5-b) pyridine (CID 1530); (**b**) isoquercitrin (CID 5280804); (**c**) belzutifan (CID 117947097); (**d**) levofloxacin (CID 149096). (**B**) (**a**) IL6 with isoquercitrin (CID 5280804); (**b**) belzutifan (CID 117947097); (**c**) levofloxacin (CID 149096). (**C**) MTOR with glycocholic acid (CID 10140); belzutifan (CID 117947097); levofloxacin (CID 149096). (**D**) PIK3CA with 11-methoxycurvularin (**a**) (CID 10381440); (**b**) glycocholic acid (CID 10140); (**c**) isoquercitrin (CID 5280804); (**d**) belzutifan (CID 117947097); (**e**) levofloxacin (CID 149096). (**E**) PTGS2 with (**a**) isoquercitrin (CID 5280804), (**b**) belzutifan (CID 117947097), and (**c**) levofloxacin (CID 149096).

Then, the results were visualized using Discovery Studio (Figure 5). We observed the 3D interaction modes of SCFA microbial metabolites with the kidney cancer and inflammation proteins targets.

We predict that the IGF1R target interacted with 2-amino-1-methyl-6-phenylimidazo(4,5-b) pyridine via MET1082 (hydrogen bond), as well as LEU1005, VAL1013, LYS1033, and MET1156 (other bonds), and showing a binding affinity of −7.4 kcal/mol. Another compound, isoquercitrin interacted via MET1082, MET1156, and THR1083 (hydrogen bond), as well as GLY1085, ARG1084, LEU1005, and SER1089 (other bonds), and with a binding affinity of −7.9 kcal/mol. Meanwhile, belzutifan (control drug for kidney cancer) interacted via SER1089 (hydrogen bond), as well as LEU1005, ASP1086, MET1142, MET1156, and VAL1013 (other bonds), with a binding affinity of −7.3 kcal/mol. Levofloxacin (control drug for kidney inflammation) interacted via LEU1005 (hydrogen bond), as well as MET1156, VAL1013, and ILE1160 (other bonds), and also with a binding affinity of −7.6 kcal/mol (Figure 5A, Table 3, Supplementary Figure S1A).

Then, the IL6 target interacted with isoquercitrin via ALA177, GLN114, GLY43, and THR44 (hydrogen bond), as well as VAL94, GLU157, and PRO158 (other bonds), and with a binding affinity of −7.9 kcal/mol, whereas both controls drugs showed the same binding affinity. Such as, belzutifan (control drug for kidney cancer) interacted via ARG103, LEU4, SER64, and TRP49 (hydrogen bond), as well as ALA99, LEU47, ARG103, and VAL100 (other bonds), and with a binding affinity of −7.5 kcal/mol, and levofloxacin (control drug of kidney inflammation) interacted via HIS173 and LEU139 (hydrogen bond), as well as SER141, GLY171, and PHE175 (other bonds), and with a binding affinity of −7.5 kcal/mol (Figure 5B, Table 3, Supplementary Figure S1B).

Moreover, MTOR target interacted with glycocholic acid via TYR82 (hydrogen bod) with a binding affinity of −6.5 kcal/mol, whereas, both controls drugs showed different binding affinity. Such as, belzutifan (control drug for kidney cancer) interacted via GLU54 (hydrogen bond), as well as PHE46 (other bonds), with a binding affinity of −7.4 kcal/mol, and levofloxacin (control drug of kidney inflammation) interacted via GLU54 (hydrogen bond), as well as TYR82, ILE56, and VAL55 (other bonds), with a binding affinity of −6.3 kcal/mol (Figure 5C, Table 3, Supplementary Figure S1C).

In addition, PIK3CA target interacted with 11-methoxycurvularin via GLN682 and SER464 (hydrogen bond), as well as VAL680, GLU135, and PRO466 (other bonds), with a binding affinity of −8.2 kcal/mol. Then, glycocholic acid interacted with MET811, GLU259, and ASP258 (hydrogen bod) with a binding affinity of −9.3 kcal/mol. Finally, isoquercitrin interacted via GLN682, LYN678, SER464, ASN428 (hydrogen bond), and ASP133 (other bonds) with a binding affinity of −8.4 kcal/mol, whereas belzutifan (control drug for kidney cancer) interacted via ARG818 and HIS670 (hydrogen bond), as well as SER629, PHE666, and LEU755 (other bonds) with a binding affinity of −7.3 kcal/mol, and levofloxacin (control drug of kidney inflammation) interacted via ARG683 and ASN428 (hydrogen bond), as well as GLU135, LEU645, and GLN643 (other bonds), with a binding affinity of −8.1 kcal/mol (Figure 5D, Table 3, Supplementary Figure S1D).

Finally, PTGS2 target interacted with isoquercitrin via TYR136 and ASN34 (hydrogen bond), as well as PRO156, CYS36, PRO154, and ASP157 (other bonds), with a binding affinity of −9.5 kcal/mol, whereas belzutifan (control drug for kidney cancer) interacted via ARG376, GLN375, and ASN376 (hydrogen bond), as well as LEU224, GLY225, and PHE142 (other bonds), with a binding affinity of −9.1 kcal/mol. Levofloxacin (control drug of kidney inflammation) interacted via CYS39 and CYS47 (hydrogen bond), as well as PRO156, TYR136, PRO154, and GLN328 (other bonds), with a binding affinity of −9 kcal/mol (Figure 5E, Table 3, Supplementary Figure S1E).

## 4. Discussion

According to epidemiological data for kidney cancer (KC) as a whole, renal cell carcinoma (RCC) represents the vast majority (90%) of KC cases, with clear cell RCC (ccRCC; 70%), papillary RCC (pRCC; 10–15%), and chromophobe RCC (5%) being the most common types [49]. Recently, research topics like kidney cancer are a major challenge for scientists. To address challenges like the lack of efficacy and the development of resistance to single-targeted drugs, drug discovery frequently necessitates a system-level pharmacology approach. Network pharmacology techniques are created and used more frequently to identify new therapeutic possibilities and repurpose current medications [50]. To assess the pharmacological significance of the primary target discovered using microbiome analysis, we conducted a network pharmacology inquiry. Using data-driven analysis, we investigated the interaction of kidney cancer and inflammation with gut microbiome metabolites. Malnutrition, hypertension or hypotension, microinflammation, immune system dysbiosis, and numerous oxidative stresses are frequently associated with kidney illness and can all be treated with SCFAs [14]. SCFA-producing microbes also release different types of metabolites. However, currently, there is a lack of information on the role and interaction of gene targets with SCFA-producing microbial metabolites in regulating kidney cancer and inflammation. To investigate such a novel hypothesis, we performed several network pharmacology-based analyses. In this study, we analyzed SCFA-producing microbial metabolites and disease-related targets to investigate SCFA-producing microbial metabolites and disease networks.

During data collection, we selected microbes that produce SCFAs (177 different species, 114 metabolites, and 1890 gene targets from gutMgene [19]) and 13,104 disease targets from the DisgeNET [20], Genecard [22], and OMIM [21] database platforms. For validation of the data, we collected gene targets from three different platforms. Furthermore, we performed PPI network analysis; the physical connections between proteins in a cell were mathematically modeled by PPI networks. These unique interactions between specified binding sites in the proteins have a particular biological significance (i.e., they perform a particular function) [51]; therefore, we found functionally correlated genes. For further validation, we observed the RNA expression in different organs, and found a positive expression in the urinary bladder and kidney (Figure 4E). Absorption, distribution, metabolism, and excretion are together referred to as ADME. This group of characteristics is essential for a drug molecule to be an acceptable drug candidate within the human body. The ADME profile is affected by a number of variables, including physicochemical qualities, protein binding, solubility, permeability, and inhibitory screening results. A drug’s probability of success will also be significantly influenced by its ADME profile [52]. As a result, ADME features were used to select potentially significant substances. Finally, we performed a molecular docking simulation to predict possible targets. Due to its capacity to predict the binding conformation of small molecule ligands to the proper target binding site, molecular docking is one of the most widely utilized techniques in structure-based drug design. The rational design of medications and understanding of the basic biological processes both benefit greatly from the characterization of the binding behavior [53,54]. Therefore, after PPI network analysis, RNA expression validation, ADMET properties screening, and molecular docking simulation, we selected five (5) different targets (IGF1R, IL6, MTOR, PIK3CA, and PTGS2) and four (4) different metabolites including different targets (isoquercitrin, glycocholic acid, 11-methoxycurvularin, and 2-amino-1-methyl-6-phenylimidazo (4,5-b) pyridine). Among our selected metabolites, isoquercitrin prevents the spread of bladder, pancreatic, and liver cancer. The main mechanisms are that isoquercitrin activates caspase-3, -8, and -9; reduces the phosphorylation of ERK; and promotes the phosphorylation of c-Jun N-terminal kinase (JNK). Additionally, isoquercitrin blocks the cell cycle in the G1 phase to promote the death of cancer cells through apoptosis [55,56,57]. Further, isoquercitrin dramatically reduces the mRNA expression of proinflammatory factors such as tumor necrosis factor-, interleukin (IL)-1, IL-6, monocyte chemoattractant protein-1, and prostaglandin E synthase 2 (PTGES2). As a result, isoquercitrin serves as a possible pharmaceutical substitute for the treatment of diseases caused by inflammation [58]. Through our network pharmacology study, we found that the isoquercitrin compound can be isolated from *Bacillus sp.*, *Bacteroides sp.*, and *Eubacterium ramulus* [19] SCFA-producing microbes, and can be used to treat kidney cancer and inflammation-related diseases by regulating PIK3CA, IL6, PTGS2, and IGF1R gene targets (Table 4). Another compound, glycocholic acid, was discovered to be a highly effective and secure anti-inflammatory medication. It is a lead compound that can be utilized to treat an overactive immune system [59]. We also found that glycocholic acid can be isolated from *Bacteroides fragilis*, *Butyricicoccus pullicaecorum*, and *Ruminococcus flavefaciens* [19] SCFA-producing microbes, and can be used to treat kidney cancer and inflammation-related diseases through regulating MTOR and PIK3CA targets (Table 4). Furthermore, 11-methoxycurvularin derived from a fungal strain *Penicillium* sp. and acts as an anti-inflammatory compound that exhibits strong inhibitory effects on nitric oxide (NO) and prostaglandin E_2_ (PGE_2_), with IC_50_ values ranging from 1.9 to 18.1μm, and also on IC_50_ values from 2.8 to 18.7 μM, respectively, in RAW264.7 cells induced by LPS [60]. In our study, we found that the 11-methoxycurvularin compound could be isolated from *Bacillus sp.* SCFAs produce microbes [19] and can be used to treat kidney cancer and inflammation-related diseases through regulating MTOR and PIK3CA targets (Table 4). Finally, pathway analysis and gene−gene interactions show that 2-amino-1-methyl-6-phenylimidazo[4,5-b] pyridine (PhIP) regulates STAT3-regulated genes and starts leptin signaling through the JAK/STAT and MAPK pathway cascades. PhIP can be isolated from *Blautia obeum*, *Faecalibacterium prausnitzii*, and *Lactobacillus reuteri* SCFA microbes [19]. As a result of the many limitations and toxicity of this compound, it cannot be used as a postbiotic compound [61]. However, further experiments need to be conducted to confirm the role and relation of these compounds with the IGF1R target of kidney inflammation and kidney cancer.

## 5. Conclusions

Our findings imply that a variety of microorganisms can offer crucial metabolites against kidney cancer and disorders linked to inflammation. We found four typical microbial metabolites along with five gene targets related to kidney cancer and inflammatory diseases. SCFA-producing microbial metabolites, such as isoquercitrin, 11-methoxycurvularin, and glycocholic acid, are involved in regulating kidney cancer and inflammation-related diseases, and could be useful as postbiotics. Moreover, isoquercitrin with PIK3CA, IL6, PTGS2, and IGF1R gene targets; 11-methoxycurvularin; and glycocholic acid with MTOR and PIK3CA gene targets are all involved in controlling kidney cancer and diseases related to kidney inflammation.

However, there are certain limitations on the amount of information that has been assembled on the microbiome. Because of the limitations of bioinformatics and chem-informatics, we propose conducting additional preclinical or clinical experiments to confirm these findings.

## Figures and Tables

**Table 1:** Degree centrality (DC), betweenness centrality (BC), and closeness centrality (CC) of kidney cancer and kidney inflammation genes.

Name	Betweenness Centrality	Closeness Centrality	Degree	Number of Directed Edges
TP53	0.319071552	0.872340426	35	2
CTNNB1	0.108958047	0.732142857	26	20
IL6	0.156313791	0.683333333	23	23
MTOR	0.054536656	0.672131148	22	5
PTGS2	0.064988518	0.650793651	20	35
PIK3CA	0.03684383	0.630769231	18	17
ERBB2	0.0341558	0.630769231	17	10
NFKBIA	0.010607996	0.594202899	14	26
KEAP1	0.012401937	0.585714286	13	7
SIRT1	0.020993867	0.585714286	13	6
RELA	0.003634516	0.569444444	11	18
GSTP1	0.01601626	0.554054054	11	22
MET	0.005245046	0.561643836	10	4
SLC2A1	0.007293932	0.561643836	10	3
IGF1R	0.000513	0.554054054	9	6
VHL	0.003383222	0.525641026	8	7
LRRK2	0.006013884	0.532467532	8	14
CTSD	0.006634727	0.518987342	8	8
CYP1A1	0.005259476	0.539473684	7	1
FLT1	0.001178862	0.5125	7	1

**Table 2:** Microbial metabolites and hub targets after ADME property analysis.

Hub Target Genes	Gene Targeted Compounds and ID	Target Microbes [19]
TP53	Bile acid (439520, CHEBI:22868)	*Bacteroides distasonis*, *Clostridium scindens*, *Faecalibacterium prausnitzii*, *Haemophilus parainfluenzae.*
CTNNB1	2-Hydroxy-3-(5-hydroxy-1H-indol-3-yl)propanoic acid (192215)	*Clostridium sporogenes.*
	3-Hydroxy-4-methoxybenzenepropanoic acid (2752054, HMDB0131138)	*Clostridium orbiscindens*, *Eubacterium ramulus*,
	Dihydrocaffeic acid (348154, HMDB0000423, CHEBI:48400)	*Bifidobacterium*, *Bifidobacterium longum*, *Clostridium orbiscindens*, *Clostridium sporogenes*, *Eubacterium ramulus*, *Faecalibacterium prausnitzii*, *Lactobacillus mucosae*, *Lactobacillus zeae.*
	Indole-3-lactic acid (92904, CHEBI:24813)	*Clostridium sporogenes.*
MTOR	11-Methoxycurvularin (10381440)	*Bacillus* sp.
	Dihydrodaidzein (176907, HMDB0005760, CHEBI:75842)	*Blautia producta*, *Bacillus* sp., *Clostridium* sp., *Lactobacillus mucosae*, *Lactococcus* sp.,
	Glycocholic acid (10140, HMDB0000138, CHEBI:17687)	*Bacteroides fragilis*, *Butyricicoccus pullicaecorum*, *Ruminococcus flavefaciens.*
PIK3CA	11-Methoxycurvularin (10381440)	*Bacillus* sp.
	3-Hydroxyphenethyl alcohol (83404)	*Bifidobacterium.*
	Caffeic acid (689043, HMDB0001964, CHEBI:16433)	*Bifidobacterium*, *Bifidobacterium animalis.*
	Dihydrodaidzein (176907, HMDB0005760, CHEBI:75842)	*Blautia producta*, *Bacillus* sp., *Clostridium* sp., *Lactobacillus mucosae*, *Lactococcus* sp.,
	Dihydroglycitein (101101166, CHEBI:174736)	*Eubacterium limosum.*
	Glycocholic acid (10140, HMDB0000138, CHEBI:17687)	*Bacteroides fragilis*, *Butyricicoccus pullicaecorum*, *Ruminococcus flavefaciens.*
	Isoquercitrin (5280804, HMDB0037362, CHEBI:68352)	*Bacillus* sp., *Bacteroides* sp., *Eubacterium ramulus.*
IL6	Acetate (175, CHEBI:30089)	*Bacteroides thetaiotaomicron*, *Bacteroidetes*, *Bifidobacterium dentium*, *Bifidobacterium longum*, *Blautia faecis*, *Clostridium asparagiforme*, *Clostridium pasteurianum*, *Clostridium scindens*, *Clostridium sp. L2-50*, *Eubacterium limosum*, *Eubacterium ramulus*, *Eubacterium rectale*, *Lawsonibacter asaccharolyticus*, *Ruminococcus champanellensis*, *Succinivibrio dextrinosolvens*,
	Butyrate (104775, CHEBI:17968)	*Butyricimonas synergistica*, *Butyricimonas virosa*, *Clostridium*, *Clostridium butyricum*, *Clostridium pasteurianum*, *Clostridium tyrobutyricum*, *Eubacterium hallii*, *Eubacterium limosum*, *Eubacterium ramulus*, *Eubacterium rectale*, *Faecalibacterium prausnitzii*, *Firmicutes*, *Fusobacteriia*, *Lawsonibacter asaccharolyticus*, *Prevotella copri*, *Roseburia inulinivorans.*
	Isoquercitrin (5280804, HMDB0037362, CHEBI:68352)	*Bacillus* sp., *Bacteroides* sp., *Eubacterium ramulus.*
	Propionate (104745, CHEBI:17272)	*Bacteroides*, *Bacteroides thetaiotaomicron*, *Eubacterium limosum*, *Haemophilus parainfluenzae*, *Parasutterella excrementihominis*, *Phascolarctobacterium succinatutens*, *Propionibacterium avidum*, *Roseburia inulinivorans*, *Ruminococcus bromii*, *Veillonella*, *Veillonella ratti.*
ERBB2	2,3-Dihydroxypropyl (*E*)-3-(3,4-dihydroxyphenyl)prop-2-enoate (5315606)	*Bifidobacterium.*
	2-Hydroxy-3-(5-hydroxy-1H-indol-3-yl)propanoic acid (192215)	*Clostridium sporogenes.*
	3-Hydroxy-4-methoxybenzenepropanoic acid (2752054, HMDB0131138)	*Clostridium orbiscindens*, *Eubacterium ramulus.*
	4-Hydroxy-(3′,4′-dihydroxyphenyl)-valeric acid (52920332, HMDB0041679, CHEBI:137478)	*Lactobacillus plantarum.*
	5-(3,4-Dihydroxyphenyl)-valerolactone (45093073)	*Lactobacillus plantarum.*
	Caffeic acid (689043, HMDB0001964, CHEBI:16433)	*Bifidobacterium*, *Bifidobacterium animalis.*
	Ethyl phenyllactate, (-)- (9877619, HMDB0032618)	*Bacteroides caccae*, *Clostridium* sp.
	Hydroquinone (785, HMDB0002434, CHEBI:17594)	*Bacteroides*, *Bifidobacterium*, *Bifidobacterium longum*, *Eubacterium.*
	Indole-3-lactic acid (92904, CHEBI:24813)	*Clostridium sporogenes.*
PTGS2	(*R*)-3-(4-Hydroxyphenyl)lactate (9548632, CHEBI:10980)	*Bacteroides caccae*, *Clostridium* sp.
	2-(4-Hydroxyphenyl)propionic acid, (2*S*)- (6971268)	*Eubacterium ramulus.*
	2,3-Dihydroxypropyl (*E*)-3-(3,4-dihydroxyphenyl)prop-2-enoate (5315606)	*Bifidobacterium*,
	2-Hydroxy-3-(4-hydroxyphenyl)propanoic acid (9378, HMDB0000755, CHEBI:17385)	*Clostridium sporogenes.*
	2-Hydroxy-3-(5-hydroxy-1H-indol-3-yl)propanoic acid (192215)	*Clostridium sporogenes.*
	3-(3,4-Dihydroxyphenyl)-2-hydroxypropanoic acid (439435, HMDB0003503, CHEBI:17807)	*Clostridium sporogenes.*
	3-(3-Hydroxyphenyl)propanoic acid (91, HMDB0000375, CHEBI:1427)	*Bifidobacterium.*
	3-(4-Hydroxyphenyl)propionic acid (10394, HMDB0002199, CHEBI:32980)	*Clostridium orbiscindens*, *Eubacterium ramulus.*
	3,4-Dihydroxybenzoic acid (72, CHEBI:36062)	*Bacteroides* sp.
	3,4-Dihydroxyphenylacetic acid (547, HMDB0001336, CHEBI:41941)	*Clostridium orbiscindens*, *Eubacterium ramulus*,
	3-Hydroxy-4-methoxybenzenepropanoic acid (2752054, HMDB0131138)	*Clostridium orbiscindens*, *Eubacterium ramulus*,
	3-Hydroxybenzoic acid (7420, HMDB0002466, CHEBI:30764)	*Eubacterium.*
	3-Hydroxyphenethyl alcohol (83404)	*Bifidobacterium.*
	3-Phenylpropionic acid (107, CHEBI:28631)	*Clostridium sporogenes*
	4-Hydroxy-(3′,4′-dihydroxyphenyl)-valeric acid (52920332, HMDB0041679, CHEBI:137478)	*Lactobacillus plantarum.*
	4-Hydroxybenzoic acid (135, HMDB0000500, CHEBI:30763)	*Eubacterium.*
	4-Hydroxyphenylacetic acid (127, HMDB0000020, CHEBI:18101)	*Eubacterium ramulus*,
	Caffeic acid (689043, HMDB0001964, CHEBI:16433)	*Bifidobacterium*, *Bifidobacterium animalis.*
	Dihydrocaffeic acid (348154, HMDB0000423, CHEBI:48400)	*Bifidobacterium*, *Bifidobacterium longum*, *Clostridium orbiscindens*, *Clostridium sporogenes*, *Eubacterium ramulus*, *Faecalibacterium prausnitzii*, *Lactobacillus mucosae*, *Lactobacillus zeae.*
	D-Lactic acid (61503, HMDB0001311, CHEBI:42111)	*Faecalibacterium prausnitzii*
	Ethyl phenyllactate, (-)- (9877619, HMDB0032618)	*Bacteroides caccae*, *Clostridium* sp.
	Isobutyric acid (6590, HMDB0001873, CHEBI:16135)	*Butyricimonas synergistica*, *Butyricimonas virosa*
	Isoquercitrin (5280804, HMDB0037362, CHEBI:68352)	*Bacillus* sp., *Bacteroides* sp., *Eubacterium ramulus.*
	Leucine (6106, HMDB0000687, CHEBI:15603)	*Blautia*, *Faecalibacterium prausnitzii*, *Ruminococcus*
	Phenolic acid (CHEBI:166890)	*Eubacterium ramulus.*
	Phenylacetic acid (999, HMDB0000209, CHEBI:30745)	*Bifidobacterium.*
	Pipecolic acid (849, HMDB0000070, CHEBI:17964)	*Lactobacillus casei.*
	Proline (145742, HMDB0000162, CHEBI:17203)	*Blautia*, *Ruminococcus.*
	Quinic acid (6508, HMDB0003072, CHEBI:17521)	*Bifidobacterium animalis.*
IGF1R	2-Amino-1-methyl-6-phenylimidazo[4,5-b] pyridine (1530, CHEBI:76290)	*Blautia obeum*, *Faecalibacterium prausnitzii*, *Lactobacillus reuteri.*
	3-(3,4-Dihydroxyphenyl)-2-hydroxypropanoic acid (439435, HMDB0003503, CHEBI:17807)	*Clostridium sporogenes*
	3-(3-Hydroxyphenyl)propanoic acid (91, HMDB0000375, CHEBI:1427)	*Bifidobacterium.*
	3-(4-Hydroxyphenyl)propionic acid (10394, HMDB0002199, CHEBI:32980)	*Clostridium orbiscindens*, *Eubacterium ramulus.*
	3,4-Dihydroxybenzoic acid (72, CHEBI:36062)	*Bacteroides* sp.
	3,4-Dihydroxyphenylacetic acid (547, HMDB0001336, CHEBI:41941)	*Clostridium orbiscindens*, *Eubacterium ramulus*,
	3-Hydroxy-4-methoxybenzenepropanoic acid (2752054, HMDB0131138)	*Clostridium orbiscindens*, *Eubacterium ramulus*,
	3-Hydroxybenzoic acid (7420, HMDB0002466, CHEBI:30764)	*Eubacterium.*
	4-Hydroxy-(3′,4′-dihydroxyphenyl)-valeric acid (52920332, HMDB0041679, CHEBI:137478)	*Lactobacillus plantarum.*
	4-Hydroxybenzoic acid (135, HMDB0000500, CHEBI:30763)	*Eubacterium.*
	Dihydrocaffeic acid (348154, HMDB0000423, CHEBI:48400)	*Bifidobacterium*, *Bifidobacterium longum*, *Clostridium orbiscindens*, *Clostridium sporogenes*, *Eubacterium ramulus*, *Faecalibacterium prausnitzii*, *Lactobacillus mucosae*, *Lactobacillus zeae.*
	Dihydrodaidzein (176907, HMDB0005760, CHEBI:75842)	*Blautia producta*, *Bacillus* sp., *Clostridium* sp., *Lactobacillus mucosae*, *Lactococcus* sp.,
	Ethyl phenyllactate, (-)- (9877619, HMDB0032618)	*Bacteroides caccae*, *Clostridium* sp.
	Glutathione (124886, HMDB0000125, CHEBI:16856)	*Bacteroides thetaiotaomicron.*
	Isoquercitrin (5280804, HMDB0037362, CHEBI:68352)	*Bacillus* sp., *Bacteroides* sp., *Eubacterium ramulus.*
	Phenolic acid (CHEBI:166890)	*Eubacterium ramulus.*
RELA	2,3-Dihydroxypropyl (E)-3-(3,4-dihydroxyphenyl)prop-2-enoate (5315606)	*Bifidobacterium.*
	Caffeic acid (689043, HMDB0001964, CHEBI:16433)	*Bifidobacterium*, *Bifidobacterium animalis.*

**Table 3:** Molecular docking simulation between five targets and their associated compounds.

Targets	Compound	Binging Energy	Hydrogen Bond	Other Bonds	Grid Box Center	Dimension
IGF1R	2-Amino-1-methyl-6-phenylimidazo(4,5-b) pyridine_CID_1530	−7.4	MET	LEU, VAL, LYS, MET	x= 20.89, y = 3.76, z= 45.48	x = 74.703, y = 55.85, z = 62.013
	Isoquercetin_COMPOUND_CID_5280804	−7.9	MET, THR	GLY, ARG, LEU, SER		
	Control_Belzutifan_Cancer_CID_117947097	−7.3	SER	LEU, ASP, MET, VAL		
	Control_levofloxacin__inflammation_CID_149096	−7.6	LEU	MET, VAL, ILE		
IL6	Isoquercetin_CID_5280804	−7.9	ALA, GLN, GLY, THR	VAL, GLU, PRO	x = 16.06, y = 14.52, z = 22.37	x = 64.69, y = 83.398, z = 56.699
	Belzutifan_Cancer_CID_117947097	−7.5	ARG, LEU, SER, TRP	ALA, LEU, ARG, VAL		
	levofloxacin__inflammation_CID_149096	−7.5	HIS, LEU	SER, GLY, THR		
MTOR	2fap_Glycocholic acid_COMPOUND_CID_10140	−6.5	TYR		x = −6.45, y = 21.63, z = 43.68	x = 35.723, y = 45.28, z = 33.53
	2fap_Belzutifan_Cancer_COMPOUND_CID_117947097	−7.4	GLU	PHE		
	2fap_levofloxacin__inflammation_COMPOUND_CID_149096	−6.3	GLU	TYR, ILE, VAL		
PIK3CA	5dxt.p_11-Methoxycurvularin_COMPOUND_CID_10381440	−8.2	GLN, SER	VAL, GLU, PRO	x = −1.83, y = 5.71, z = 17.04	x = 79.48, y = 96.48, z = 89.84
	5dxt.p_Glycocholic acid_COMPOUND_CID_10140	−9.3	MET, GLU, ASP			
	5dxt.p_Isoquercetin_CID_5280804	−8.4	GLN, LYN, SER, ASN	ASP		
	5dxt.p_Belzutifan__CID_117947097	−7.3	ARG, HIS	SER, PHE, LEU		
	5dxt.p_levofloxacin__inflammation_COMPOUND_CID_149096	−8.1	ARG, ASN	GLU, LEU, GLN		
PTGS2	5ikq.pp_Isoquercetin_COMPOUND_CID_5280804	−9.5	TYR, ASN	PRO, CYS, ASP	x = 27.15, y = 38.38, z= 41.79	x = 82.98, y = 83.28, z = 104.06
	5ikq.pp_Belzutifan_Cancer_COMPOUND_CID_117947097	−9.1	ARG, GLN, ASN	LEU, GLY, PHE		
	5ikq.pp_levofloxacin__inflammation_COMPOUND_CID_149096	−9	CYS	PRO, TYR, GLN		

**Table 4:** Final targets, compounds, and microbial sources for kidney cancer and inflammation-related diseases.

Final Target Gene	Gene Targeted Compounds and ID	Target Microbes [19]
MTOR	11-Methoxycurvularin (10381440)	*Bacillus* sp.
	Glycocholic acid (10140, HMDB0000138, CHEBI:17687)	*Bacteroides fragilis*, *Butyricicoccus pullicaecorum*, *Ruminococcus flavefaciens.*
PIK3CA	11-Methoxycurvularin (10381440)	*Bacillus* sp.
	Glycocholic acid (10140, HMDB0000138, CHEBI:17687)	*Bacteroides fragilis*, *Butyricicoccus pullicaecorum*, *Ruminococcus flavefaciens.*
	Isoquercitrin (5280804, HMDB0037362, CHEBI:68352)	*Bacillus* sp., *Bacteroides* sp., *Eubacterium ramulus.*
IL6	Isoquercitrin (5280804, HMDB0037362, CHEBI:68352)	*Bacillus* sp., *Bacteroides* sp., *Eubacterium ramulus.*
PTGS2	Isoquercitrin (5280804, HMDB0037362, CHEBI:68352)	*Bacillus* sp., *Bacteroides* sp., *Eubacterium ramulus.*
IGF1R	2-Amino-1-methyl-6-phenylimidazo[4,5-b] pyridine (1530, CHEBI:76290)	*Blautia obeum*, *Faecalibacterium prausnitzii*, *Lactobacillus reuteri.*
	Isoquercitrin (5280804, HMDB0037362, CHEBI:68352)	*Bacillus* sp., *Bacteroides* sp., *Eubacterium ramulus.*

## Data Availability

All data generated or analyzed during this study are included in this published article (and its Supplementary Information files).

## Supplementary Materials

The following supporting information can be downloaded at: https://www.mdpi.com/article/10.3390/biom13111678/s1. Supplementary S1. SCFAs microbe’s database (compounds and targets). Supplementary S2: Kidney cancer and inflammation database (diseases targets). Supplementary S3: GeneMANIA analysis result (suitable target genes). Supplementary S4: Molecular docking simulation results (between all selected compounds and targets). Supplementary S5: GO and KEGG pathway analysis. Supplementary S6: PPI network analysis. Top 20 target selection. Supplementary S7: ADME properties of selected compounds. Supplementary Figure S1: Molecular docking analysis of selected compounds and targets showing different bond attractions between the compounds and targets.
