# Supplementary material for: A Network Pharmacology and Molecular-Docking-Based Approach to Identify the Probable Targets of Short-Chain Fatty-Acid-Producing Microbial Metabolites against Kidney Cancer and Inflammation
Source: Biomolecules. 2023 Nov 20;13(11):1678. doi: 10.3390/biom13111678 (PMC10669250; doi:10.3390/biom13111678)
Supplement: Supplementary file 1 [file biomolecules-13-01678-s001.zip › supplementary/Supplementary Figure S1.pdf]

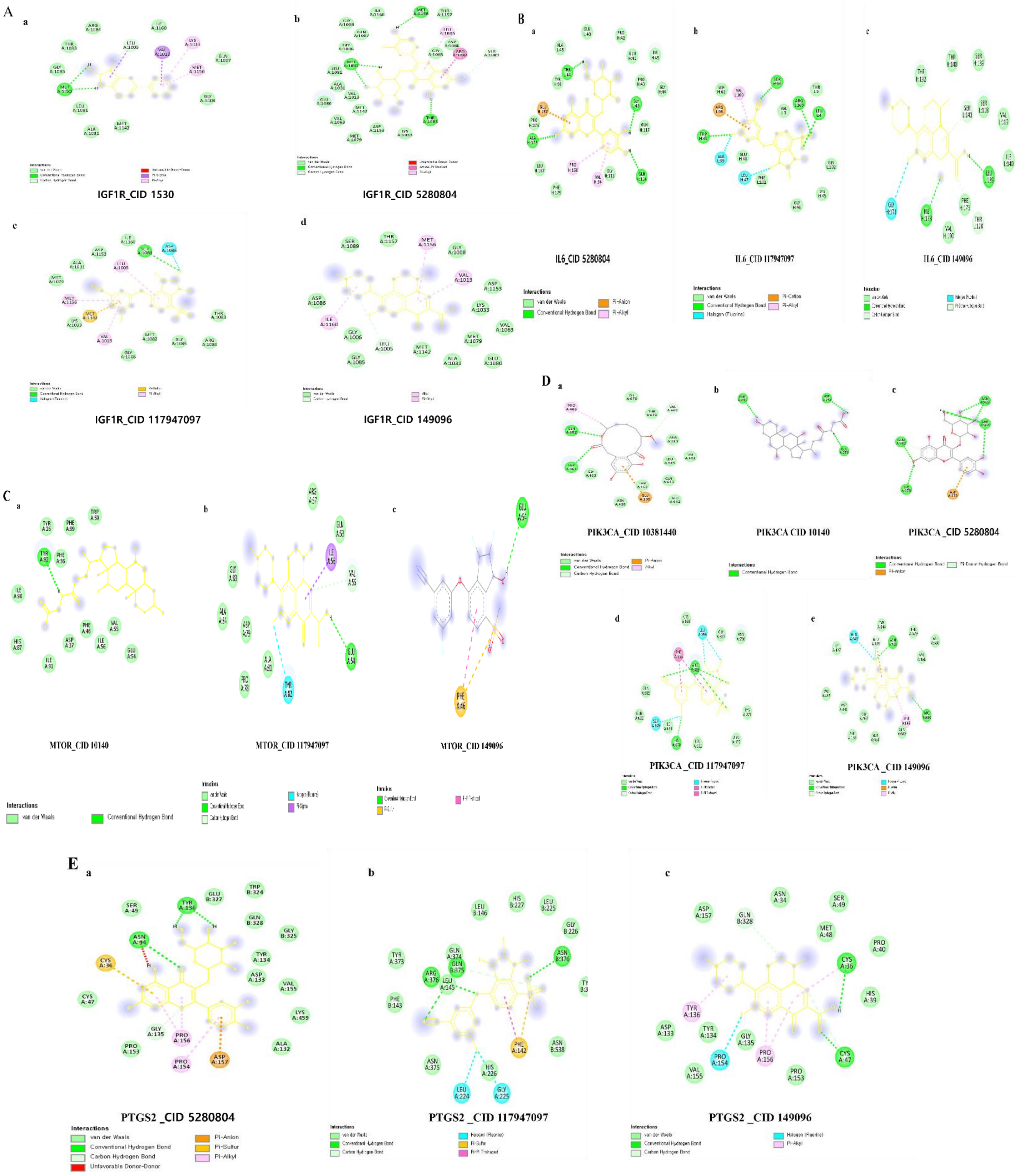

Supplementary Figure 1. Molecular docking analysis of selected compounds and targets. Showing different bond attraction between compounds and targets.
