# Supplementary material for: A Network Pharmacology and Molecular-Docking-Based Approach to Identify the Probable Targets of Short-Chain Fatty-Acid-Producing Microbial Metabolites against Kidney Cancer and Inflammation
Source: Biomolecules. 2023 Nov 20;13(11):1678. doi: 10.3390/biom13111678 (PMC10669250; doi:10.3390/biom13111678)
Supplement: Supplementary file 1 [file biomolecules-13-01678-s001.zip › supplementary/Supplementary S3. GeneMANIA analysis result (suitable target genes).pdf]

GeneMANIA report

Created on : 21 July 2023 12:45:43
Last database update : 13 August 2021 00:00:00
Application version : 3.6.0

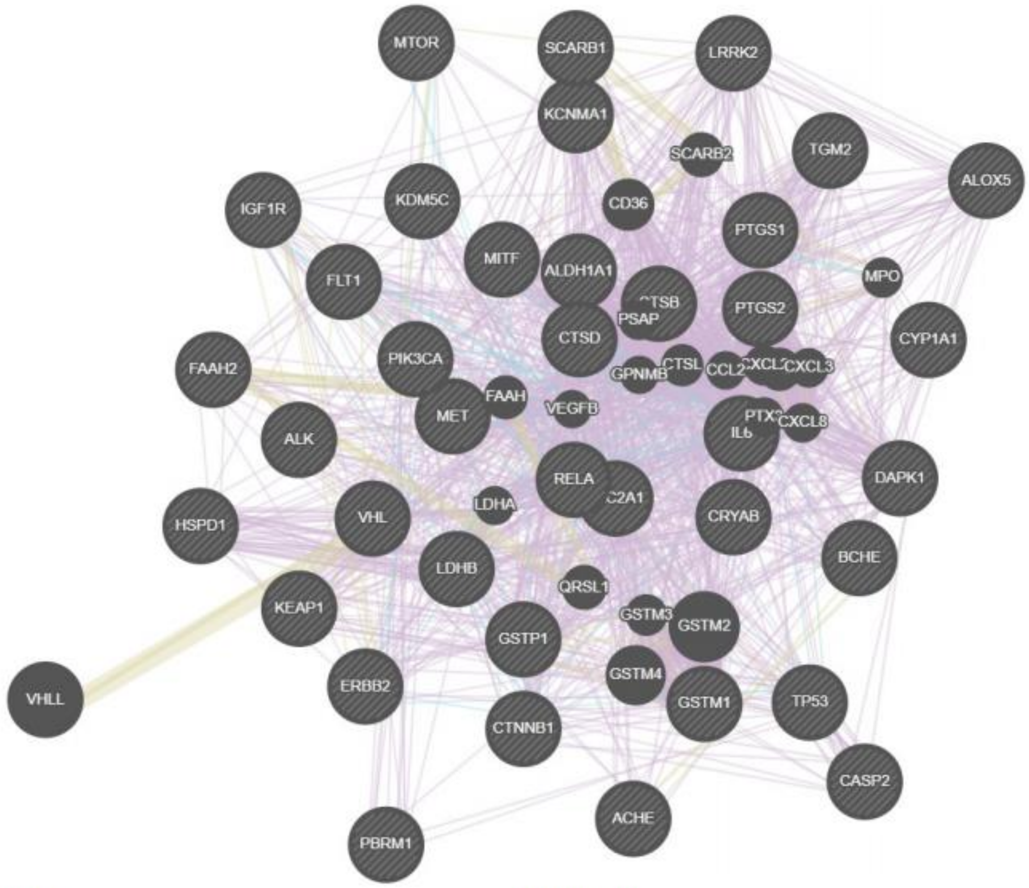

Networks

- Co-expression
- Shared protein domains
- Pathway

Functions

N/A

Search parameters

Organism Homo sapiens (human)
Genes ALOX5, SCARB1 , PTGS2 , KEAP1 , GSTP1 , ACHE , PTGS1 , FLT1 , MET , CASP2 , CRYAB , LDHB , BCHE , SLC2A1 , CTSD , PIK3CA , KDM5C , VHL , GSTM1 , IL6 , IGF1R , CYP1A1 , TP53 , ERBB2 , HSPD1 , KCNMA1 , PBRM1 , CTSB , ALDH1A1 , FAAH2 , CTNNB1 , ALK , RELA , MITF , LRRK2 , DAPK1 , MTOR , TGM2
Network weighting Automatically selected weighting method
